# Continuous laccase concentration in an aqueous two-phase system

**DOI:** 10.1007/s11696-017-0330-5

**Published:** 2017-11-11

**Authors:** Michał Blatkiewicz, Anna Antecka, Andrzej Górak, Stanisław Ledakowicz

**Affiliations:** 1 Faculty of Process and Environmental Engineering, Lodz University of Technology, ul. Wólczańska 213, 90-942 Lodz, Poland; 2 The Department of Biochemical and Chemical Engineering, Dortmund University of Technology, Emil-Figge-Straße 70, 44227 Dortmund, Germany

**Keywords:** ATPS, Model, Laccase, Downstream processing

## Abstract

An approach to describe continuous partitioning of *Cerrena unicolor* laccase in a PEG 6000—phosphate aqueous two-phase system was proposed. The laccase was separated from crude supernatant of *C. unicolor*-submerged culture, and all the experiments were carried out in 25 °C and pH 7 conditions. Masses of both phases and their compositions at phase equilibrium, as well as laccase activity concentrations at different mixing points, were measured in batch experiments. An empirical short-cut method was developed which allows for calculation of mass and volume fractions of the phases, laccase concentration factors, and laccase recoveries. Theoretical predictions were verified by several experiments carried out in a special mixer-settler unit with automatic substrate feed and continuous collection of separated phases. Required concentration of the laccase was possible to achieve in a one-step extraction process in the mixer-settler unit. The predictions of the short-cut method were compared to the results of experimental measurements of phase compositions, phase volume fractions, concentration factors and enzymatic yields at steady-state operation of the extraction unit. The values of experimental results lay well within the 10% error range of the predicted values.

## Introduction

Laccases (EC 1.10.3.2, *p*-diphenolic oxidases) are extracellular enzymes belonging to the oxidoreductase group, first discovered by Yoshida (1883). They are produced in nature by many different organisms, such as fungi (Baldrian 2006), plants (Harvey and Walker 1999), bacteria (Claus and Filip 1997), and even insects (Dittmer et al. 2004). Their major role is to metabolize durable lignocellulose chains (Cohen et al. 2002), but they can also be used to decompose various aromatic compounds, such as phenols, thiols or aromatic amines (Xu 1996), which makes them attractive in many industrial applications. Laccases can be thus used for bioremediation (Strong and Claus 2011), modification of textile fibers (Sójka-Ledakowicz et al. 2007), paper pulp delignification (Camarero et al. 2007), etc. *Cerrena unicolor*, commonly known as mossy maze polypore, is a white-rot fungus which belongs to *Polyporaceae* family, known for high secretion of laccases without the use of inductors (Rogalski and Janusz 2010).

Aqueous two-phase extraction (ATPE) is a well-established method of biomolecule concentration and purification. It allows for separation of biological products without the risk of denaturation, due to high water content in both phases and lack of non-polar solvents (Diamond 1990). An aqueous two-phase system (ATPS) consists of two phases, being aqueous solutions of mutually immiscible compounds. Aqueous two-phase systems come in a variety of constituent chemical pairs, but the most common ones include polymer–polymer (Madeira et al. 2011) and polymer–salt systems (Silvério et al. 2010). Just like classical extraction processes, ATPE can be performed in both batch and continuous mode. Several different modes of continuous ATPE have been reported, including the use of column contactors (Srinivas et al. 2002), mixer-settler units (Veide et al. 1984) or raining-bucket contactors (Jarudilokkul et al. 2000). A few review papers describing aqueous two-phase extraction properties and advances in details have been published (Raja et al. 2011; Yang et al. 2013; Iqbal et al. 2016)

Mathematical models are a useful tool for cost and effort reduction in industrial process development (Gosling 2005). Mathematical modeling of ATPE is, however, a difficult task due to high complexity of the process and multiplicity of its parameters. Phase-forming components, their concentrations, additives, pH, temperature, molecular masses and ionic strength all have strong influence on the ATPS behavior (Walter et al. 1985; Zaslavsky 1994). There are several approaches to modeling phase formation and equilibria in both polymer–polymer and polymer–salt ATPS, such as the osmotic virial expansion approach (Edmond and Ogston 1968), the Flory–Huggins approach based on the lattice theory (Flory 1942; Huggins 1942), a modified nonrandom two-liquid (NRTL) model (Sadeghi 2006) or the approach based on the Perturbed-Chain Statistical Associated Fluid Theory (PC-SAFT) (Reschke et al. 2014). Since the presented work is focused on laccase partitioning in a specified system and conditions, an experimental phase diagram was prepared for the equilibrium stage to avoid errors emerging from model simplifications.

The extracted product and its interactions with the system also have key role in partitioning (Azevedo et al. 2009; Wu et al. 2017). Therefore, all proposed models are limited in use and significantly simplified, depending on their applications. Models of macromolecule partitioning in ATPS usually rely on the Collander equation (Silvério et al. 2010) or linear solvation energy relationship (LSER) (Huddleston et al. 2004). There were also several attempts to predict extractive behavior of ATPS with the use of artificial neural networks (da Silva et al. 2014). Due to complexity of the problem, models often rely on empirical fitting (Mistry et al. 1996; Prinz et al. 2014; Mündges et al. 2015). In this paper, the predictions of the extractive system are also based on empirical approach. A similar laccase extraction model has been proposed by Prinz et al. (2014), but it regarded a different host producing the enzyme, and the partitioning was fitted according to tie-line length, without consideration of phase–volume ratio, which is an important parameter for separation efficiency (Blatkiewicz et al. 2016).

The scope of this paper is to present a short-cut method of continuous extraction of *Cerrena unicolor* laccase with a PEG 6000—phosphate aqueous two-phase system, based on data from batch-extraction experiments.

## Experimental

### Materials

#### Chemicals

l-Asparagine of > 99% purity, and 2,2′-azino-bis(3-ethylbenzothiazoline-6-sulphonic acid) (ABTS) of > 98% purity were purchased from Alfa Aesar (Karlsruhe, Germany). Dipotassium phosphate trihydrate of > 99% purity was purchased from Applichem (Darmstadt, Germany). Glucose of > 99% purity was purchased from Chempur (Piekary Śląskie, Poland). Yeast extract was purchased from Difco (Warszawa, Poland). *Malt Extract Agar* mixture, consisting of agar, microbial peptone and malt extract, manganese acetate tetrahydrate of > 99% purity, and tiamine of > 99% purity were purchased from Fluka (Buchs, Switzerland). PEG 6000 in the form of flakes, and monosodium phosphate dihydrate were purchased from Merck. Dipotassium phosphate of > 99% purity, monosodium phosphate of > 99% purity, magnesium sulphate heptahydrate of > 99% purity, copper sulphate pentahydrate of > 99% purity, iron chloride hexahydrate of > 99% purity, calcium nitrate tetrahydrate of > 99% purity, zinc nitrate hexahydrate of > 99% purity, and citric acid of > 99.9% purity were purchased from POCh (Gliwice, Poland).

#### Solutions

PEG 6000 was dissolved in distilled water for 50 wt% stock solutions. Salt stock solution was prepared in the form of phosphate buffer with pH 7 and 29 wt% of PO_4_^3−^ by mixing 411 g of K_2_HPO_4_·3H_2_O, 194 g of Na_2_HPO_4_·2H_2_O and 395 g of deionized water per 1 kg of solution. McIlvaine buffer of pH equal 4.5 was prepared by mixing stock solutions of 0.2 M Na_2_HPO_4_ and 0.1 M citric acid. The dilutions for the experiments were performed using deionized water.

### Analytics

#### Activity measurements

The enzymatic activity was measured with the ABTS assay according to Majcherczyk et al. (1999). The activity (U), was defined as the amount of the enzyme catalyzing the oxidation of one µmol of ABTS per minute. The pH was adjusted to 4.5 using McIlvaine buffer. The measurements were performed using a Multiskan FC Spectrometer from Thermo Scientific with 96-well plates. For each measurement, *V*_s_ = 50 µl of the sample was used, and its pH adjusted to 4.5 by adding 150 µl of the McIlvaine buffer. Then, 50 µl of 0.5 mM ABTS solution was added. Therefore, the volume of every measured sample was equal *V*_tot_ = 250 µl, and its optical length in the single well was equal *d* = 0.63 cm. The measurements of absorbance change *ΔE* were performed in 37 °C at 420 nm wavelength over *Δt* = 3 min. The extinction coefficient for ABTS at 420 nm wavelength is equal *ε*_420_ = 0.04321 L mmol^−1^ cm^−1^ (Prinz et al. 2012). If the sample’s activity was too high for the spectrometer’s range, it was diluted with a dilution factor *D*. All activity measurements were performed in triplicates. To calculate the enzymatic activity, the following equation was used:

1 $${\text{act}}\left[ {U \cdot L^{ - 1} } \right] = \frac{{\Delta E \cdot D \cdot V_{\text{tot}} }}{{\Delta t \cdot V_{\text{s}} \cdot d \cdot \varepsilon_{420} }}$$

#### Polymer content measures

The polymer content of phases was determined with high performance liquid chromatography. For this purpose, Agilent 1200 chromatographer was used equipped with SUPREMA 30 Å 8 × 300 mm column along with analogous 8 × 50 mm pre-column. Phosphate buffer with pH 6.8 was used as the eluent. Mixture components were identified according to their refractive index changes. All measurements were performed in triplicates.

#### Phosphate content measurements

The PO_4_^3−^ ion content was determined with ion-exchange chromatography. For this purpose, ICS-2100 chromatographer was used, equipped with IonPac AS-18-Fast 2 × 150 mm column with analogous 2 × 30 guard column. Deionized water was used as the eluent. Mixture components were identified according to conductivity changes. All measurements were performed in triplicates.

### Fungal culture

The *Cerrena unicolor* strain was initially grown on agar plates containing malt extract and mycological peptone. After 7 days of growth, the overgrown plates were homogenized with water, and the culture was moved into 14 L bioreactor, containing Lindeberg–Holm growth medium (1952) modified by Janusz et al. (2007) without the use of inductors. The liquid fermentation was conducted for 7 days in 28 °C, with 2 L/min air flow and 200 RPM stirring. Then, the supernatant was filtrated and frozen at − 20 °C.

### Experimental setup

#### Batch extraction experiments

**Fig. 1:**
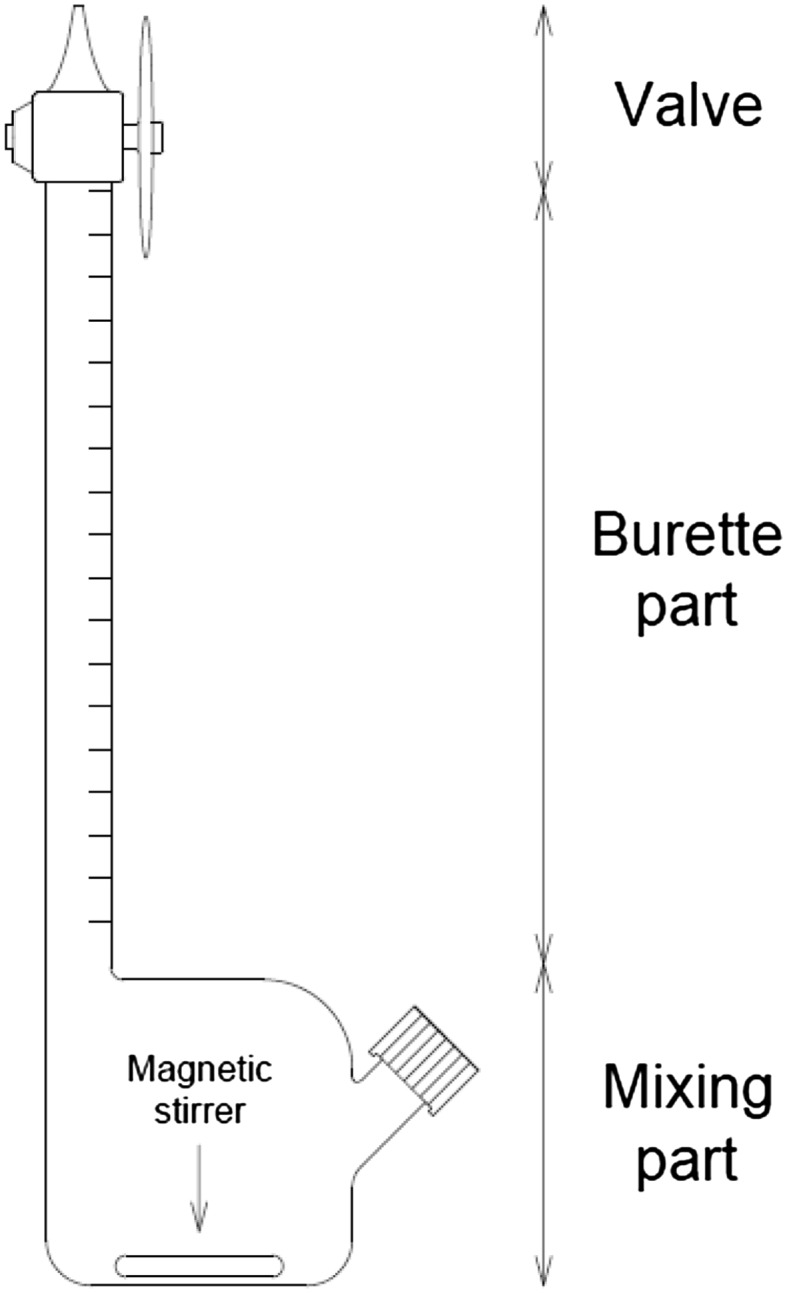
Extraction vessel

The batch extraction experiments were performed in special extraction vessels (Fig. 1), similar to those designed by Prinz et al. (2012). The vessel consisted of mixing flasks combined with burettes of approximately 20 ml volume.

Mixture components in the form of stock solutions and culture supernatant were transferred into the mixing parts of the vessel with the use of automatic pipettes, and the sample was complemented with deionized water to achieve the total mixture mass of 15 g. The mixing was carried out in a thermostated incubator equipped with a magnetic stirrer in the temperature set to 25 °C. The samples were stirred for an hour at 300 rpm to achieve thermodynamic equilibrium between the phases.

After the mixing, the stirrer was turned off and the flasks carefully flipped upside-down, to introduce the mixture into the burette part. Next, the vessels were stored in such position in 25 °C for approximately 24 h to achieve phase separation. Such long time was needed because of low-density differences and low surface tensions between the phases (Kim and Rha 2000). Finally, the flasks were removed from the incubator, and the volumes of the phases were measured according to burette indications. Phases were then collected into different vessels, and their enzymatic activities were measured.

All the extraction experiments were performed in triplicates, and the errors calculated with the use of Gaussian error propagation, considering standard deviations of experiments and systematic errors of scales, pipettes, burettes and spectrophotometric analysis.

#### Continuous extraction experiments

Continuous aqueous two-phase extraction of *C. unicolor* laccase was conducted in a mixer-settler unit (MSU), which allows for steady-state liquid flow with simultaneous phase collection. Substrate flows were set and monitored electronically with LabVision program, which controlled the flow with membrane pumps according to mass loss indications from conjugated electronic pumps. Three independent liquid streams (PEG 6000 stock solution, phosphate stock solution, *C. unicolor* culture supernatant) were thus introduced into 65 mL mixer section, where the mixture was dispersed to achieve thermodynamic equilibrium. Then, the mixture was pumped further into the settler section. The cylindrical settler part along with joint pipe from the mixer have approximately 200 mL volume. In this section, phases could settle due to very low linear flow rate of the liquid. At the other end of the settler section, both phases were collected simultaneously.

**Fig. 2:**
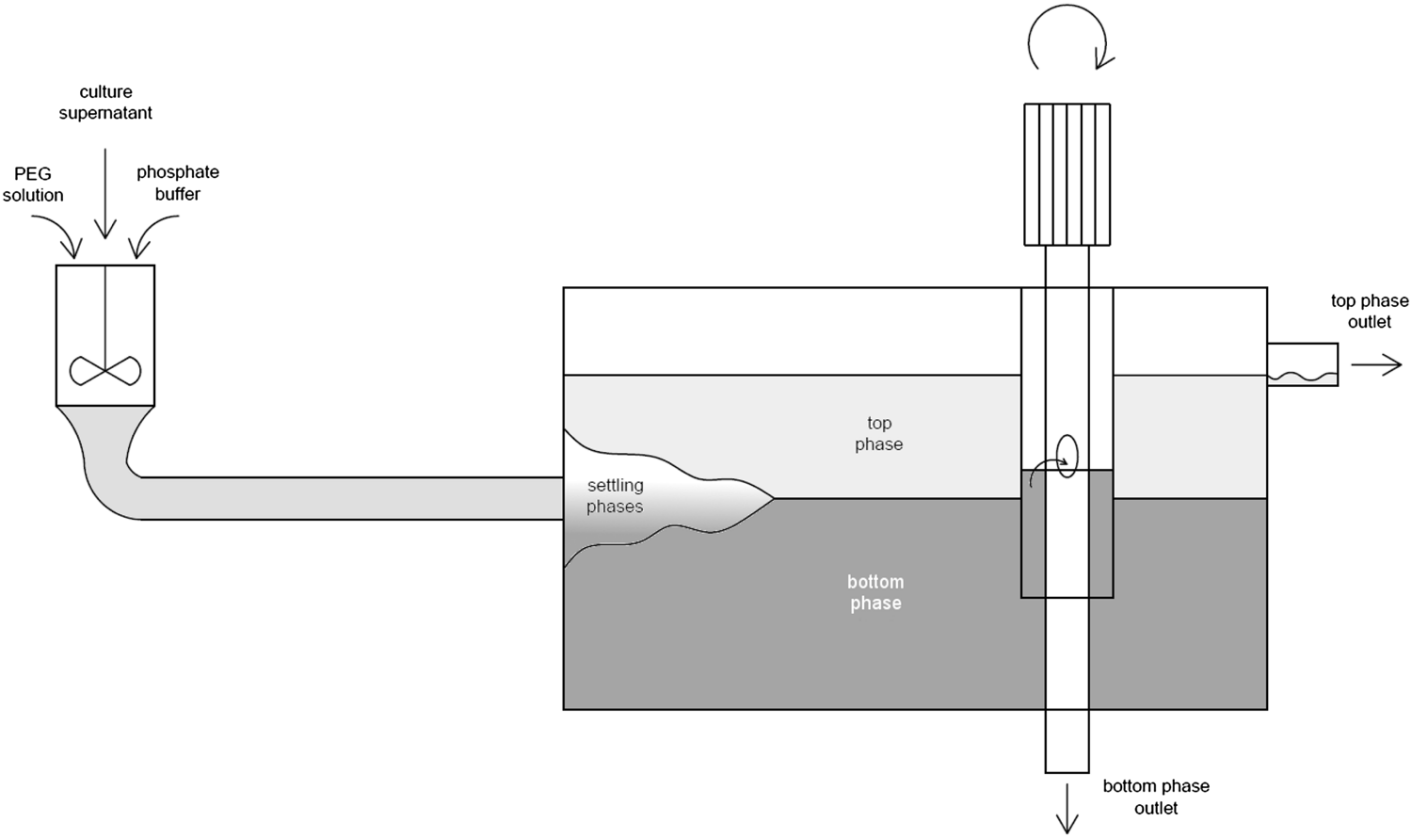
Single-stage mixer-settler unit

The settler part of the equipment was presented in Fig. 2. As long as the interface was kept above the bottom of the vertical cylinder shell, only the bottom phase was able to flow through the spillway window. At the same time, the top phase was collected through the overflow at the top of the settler section. The interphase position could be controlled with the position of the spillway window.

The separated phases flowed out of the settler towards product tanks positioned on electronic scales, the indications of which allowed for mass balance calculations. Additionally, the product outlet piping was equipped with 10 mL wells for product sampling. The amounts of products collected as samples were also considered in the mass balance. Temperature of the process was fixed at 25 °C with the use of a thermostat joined with a double-jacket system covering both the mixer and the settler sections.

First, the empty settler section was filled with dispersed mixture of PEG 6000, phosphate and *C. unicolor* culture supernatant according to the operational mixing point. Immediately after, the pump and mixer systems were launched. Both the spillway window position and the mixing rate were adjusted throughout the process to maintain a stable interphase and prevent the mixer section from overflow. After each 30 min, samples of both phases were collected, and scale indications of substrate loss and product increase were noted. The whole continuous extraction process was carried out for 5 h, after which the enzymatic activity in all the samples were immediately measured to prevent activity loss.

### Calculated parameters

The most important parameters in downstream processing are product enrichment and product recovery. Since this work concerns enrichment of laccase from crude culture supernatant, the enrichment and yield of total protein content could not represent the efficiency of the process in terms of laccase concentration. Therefore, both of these parameters are calculated with the use of enzymatic activities in the supernatant and the phases.

Product enrichment in the bottom phase (En_BP_) represents a ratio between the activity in the phase of concentration act_BP_ (here: the bottom, salt-rich phase) and the activity in the supernatant introduced into the system as substrate act_SN_.

2 $${\text{En}}_{\text{BP}} = \frac{{{\text{act}}_{\text{BP}} }}{{{\text{act}}_{\text{SN}} }}\left[ {\frac{{U \cdot L^{ - 1} }}{{U \cdot L^{ - 1} }}} \right]$$

However, the product enrichment is a parameter that does not consider dilution of the supernatant, coming from the phase-forming components and the water content in stock solutions, which vary at different mixing points. Therefore, a more general parameter called the bottom phase concentration factor (CF_BP_) was introduced, which is defined as the enrichment of the supernatant in relation to the whole volume of the system:

3 $${\text{CF}}_{\text{BP}} = \frac{{{\text{act}}_{\text{BP}} }}{{{\text{act}}_{\text{tot}} }} = \frac{{{\text{act}}_{\text{BP}} }}{{{\text{act}}_{\text{SN}} \cdot \frac{{V_{\text{SN}} }}{{V_{\text{tot}} }}}}\left[ {\frac{{U \cdot L^{ - 1} }}{{U \cdot L^{ - 1} }}} \right]$$

where *V*_SN_ stands for volume of the introduced supernatant and *V*_tot_ for total volume of the system.

The bottom phase yield (*Y*_BP_) represents the fraction of enzymatic activity recovered in a specified phase in relation to total activity introduced into the system.

4 $$Y_{\text{BP}} = \frac{{{\text{act}}_{\text{BP}} \cdot V_{\text{BP}} }}{{{\text{act}}_{\text{SN}} \cdot V_{\text{SN}} }}\left[ {\frac{U}{U}} \right]$$

where act_BP_ stands for enzymatic activity in the bottom phase, *V*_BP_ for volume of the bottom phase, act_SN_ for activity in the supernatant, and *V*_SN_ for volume of the supernatant introduced into the system.

As proven in the previous work (Blatkiewicz et al. 2016), the difference between the volumes of separated phases is a very important parameter for ATPE. Therefore, the volume fraction (VF) is also considered as one of the key parameters in this work.

5 $${\text{VF}}_{\text{BP}} = \frac{{V_{\text{BP}} }}{{V_{\text{tot}} }}\left[ {\frac{L}{L}} \right]$$

where *V*_BP_ stands for volume of the bottom phase, and *V*_tot_ for total volume of the system.

## Modeling

### Mass balance

Since amounts of substrates and phases in batch experiments are represented by their masses and volumes, while in continuous experiments they are represented by mass flow rates, the mass flow and volume flow must be converted to mass and volume, respectively. Therefore, mass flow rates of substrates and products for continuous experiments are expressed as changes of their mass over a finite period of time. To get rid of disturbances caused by product holdup in the system, the increase of mass of the product was calculated using linear regression of experimental points after the steady state was achieved. The Eq. (6) describes a linear mass increase of either one of the products or the total amount of substrates introduced into the system, where *m* stands for total mass, *ṁ* for mass flow rate expressed in [g min^−1^], Δ*t* for a specified period of time, and the index _*i*_ for the bottom phase product, top phase product, or the sum of the substrates.

6 $$m_{i} = \dot{m}_{i} \cdot \Delta t$$

Because the samples were taken each 30 min, as were noted changes in substrate and product weights, mass balances were considered in relation to a 30-min step (Eq. 7), and fulfilled the Eq. (8).

7 $$m_{i,30\hbox{ min} } = \dot{m}_{i} \cdot 30\hbox{ min}$$

8 $$m_{\text{sub}} = m_{\text{BP}} + m_{\text{TP}}$$

where sub stands for the total of substrates, BP for bottom phase and TP for top phase.

### Equilibrium stage

The design of industrial extraction equipment is based on so-called equilibrium stage, in which it is assumed that both liquid phases leaving a stage are in thermodynamic equilibrium. In this paper, this assumption was followed, and therefore, this part is focused on the modeling of phase equilibria in ATPS. While modeling, it is assumed that the phase equilibrium of the phase-building components is not affected by laccase. Therefore, the model is based on thermodynamic properties of a system consisting of PEG 6000, phosphate and water at 25 °C and pH 7.

The binodal curve was determined experimentally with mixtures consisting of PEG 6000, phosphate, and water. After setting, phase compositions were determined using chromatography, and the results were approximated with Eq. (9), similar to the one used by Prinz et al. (2014).

9 $$x_{\text{PEG}} = b_{1} \cdot x_{\text{phos}} \cdot e^{{(b_{2} \cdot x_{\text{phos}} )}} + (b_{3} - x_{\text{phos}} ) \cdot e^{{(b_{4} \cdot x_{\text{phos}} )}}$$

where *x*_PEG_ and *x*_phos_ stand for weight fractions of PEG and phosphate, respectively, and *b*_1_–*b*_4_ parameters were fitted with the least-squares method. Their values were presented in Table 1.

**Table 1:** Numerically fitted parameters for binodal curve equation

Parameter	Value
*b*_1_	14.3436
*b*_2_	− 52.5846
*b*_3_	0.6879
*b*_4_	− 90.0156

**Fig. 3:**
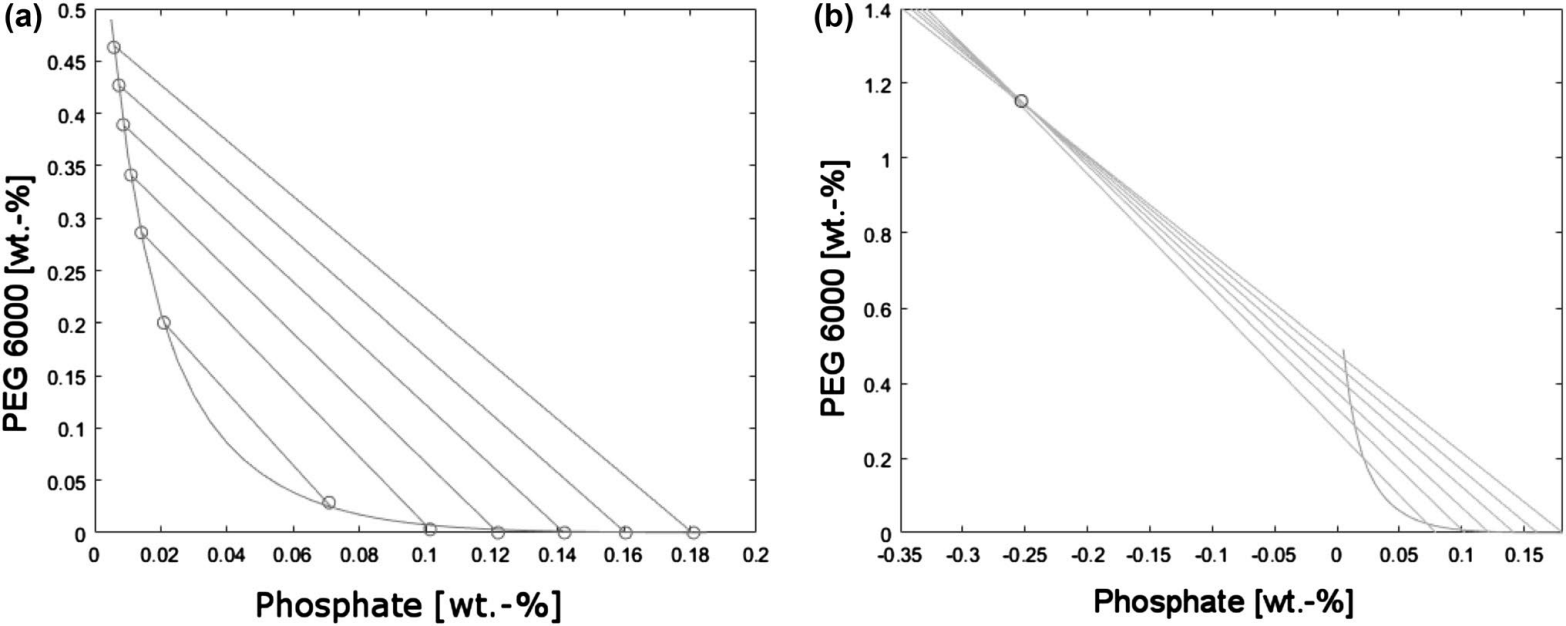
Experimental aqueous two-phase system: **a** phase diagram, **b** extended phase diagram with tie line convergence point

Tie lines connect points of phase equilibrium on the bottom phase and top phase branches and go through their respective mixing points. Using the same experimental data which was used to determine the binodal curve, a set of experimental tie lines could be drawn (Fig. 3a). It was discovered that, if extended beyond the phase diagram, the experimental tie lines crossed very closely to one common point that could be approximated (Fig. 3b). Thus, knowing the coordinates of any mixing point within the experimental range along with the tie line convergence point, a tie line can be calculated according to Eq. (10) as a straight line connecting two specified points.

10 $$x_{\text{PEG}} = \frac{{x_{\text{PEG,M}} - x_{\text{PEG,P}} }}{{x_{\text{phos,M}} - x_{\text{phos,P}} }} \cdot \left( {x_{\text{phos}} - x_{\text{phos,P}} } \right) + x_{\text{PEG,P}}$$

where *x*_PEG_ and *x*_phos_ are variables, *x*_PEG,P_ and *x*_phos,P_ are coordinates of the tie line convergence point, *x*_PEG,M_ and *x*_phos,M_ are coordinates of any mixing point. The approximated coordinates of the hypothetical tie line convergence point were presented in Table 2.

**Table 2:** Tie line convergence point coordinates

Coordinate	Value
*x*_phos,P_	− 0.253
*x*_PEG,P_	1.153

Therefore, knowing the forms of Eqs. (9) and (10), as well as the mixing point, mass fractions of both PEG and phosphate in both phases can be calculated from their solutions.

As previously stated, points describing compositions of the settled phases and the corresponding mixing points lie on one tie line. Thus, the mixing point divides the tie line |TB| into two segments: |TM| and |MB|, where *T* is the point of top phase composition, *M* is the mixing point, and *B* is the point of bottom phase composition.

In aqueous two-phase systems, just like in classical two-phase extraction systems, the lever rule obliges. It allows to calculate the mass ratios after the phase separation. For the purposes of this approach, a mass fraction of a certain phase (MF) was calculated with the lever rule, according to Eq. (11).

11 $${\text{MF}}_{i} = \frac{{m_{i} }}{{m_{\text{tot}} }} = \frac{{\sqrt {(x_{\text{phos,i}} - x_{\text{phos,M}} )^{2} + (x_{{{\text{PEG}},i}} - x_{\text{PEG,M}} )^{2} } }}{{\sqrt {(x_{\text{phos,BP}} - x_{\text{phos,TP}} )^{2} + (x_{\text{PEG,BP}} - x_{\text{PEG,TP}} )^{2} } }}$$

where the index *i* stands for top phase or bottom phase.

In many cases of multi-component mixtures, correlations between mass and density are non-linear because of the contraction effect. However, because of very low amounts of salt in polymer-rich phase and polymer in salt-rich phase, the contraction effect is negligible, and density of a phase can be calculated with Eq. (12).

12 $$\rho_{i} = \rho_{\text{PEG}} \cdot x_{{{\text{PEG,}}i}} + \rho_{S} \cdot x_{{{\text{phos}},i}} \cdot B + \rho_{{{\text{H}}_{2} {\text{O}}}} \cdot (1 - x_{{{\text{phos}},i}} \cdot B - x_{{{\text{PEG}},i}} )$$

where *ρ* stands for density [kg m^−3^] of the component and *B* is a constant parameter defined as a ratio of total mass of phosphate salt to mass of phosphate ions.

Knowing the masses of both phases from Eq. (11) and their densities from Eq. (12), it is possible to calculate the volume fraction (VF) of a phase in the system after the separation (Eq. 13).

13 $${\text{VF}}_{i} = \frac{{\frac{{m_{i} }}{{\rho_{i} }}}}{{\frac{{m_{\text{BP}} }}{{\rho_{\text{BP}} }} + \frac{{m_{\text{TP}} }}{{\rho_{\text{TP}} }}}}$$

### Empirical stage

Transport of macromolecules, such as enzymes, between phases in ATPS is a very complex process. A number of factors, including the characteristics of both the system and the extracted components influence the behavior of a biomolecule in the system. Azevedo et al. (2009) mention several such parameters, including the hydrophobicity of the molecule, the pH of the system and the average molecular size of the used polymer. The size and the isoelectric point of the extracted molecule are also key in the transport and phase equilibrium (Eiteman and Gainer 1991), but the number of factors is much larger. Wu et al. (2017) investigated 57 different physicochemical parameters of macromolecules which may influence the behavior of macromolecules in ATPS.

Because of that, the prediction of interactions between macromolecule and the system is very problematic, and any proposed thermodynamic models would be prone to very large errors. Such problems can be circumvented with the use of an empirical approach.

**Fig. 4:**
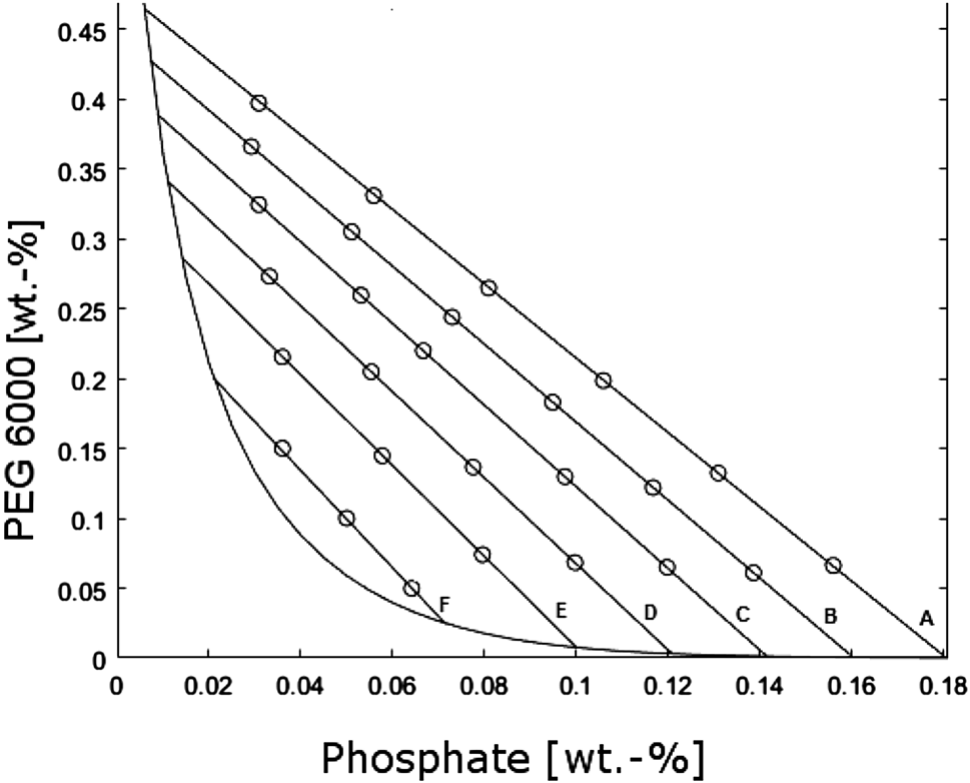
Chosen mixing points for the empirical stage

To gather the necessary data for a model, the two-phase range of the phase diagram was divided into five sectors with the experimentally determined tie lines serving as borders (Fig. 4, numbered A–F). The sector borders then were divided into sections with 27 experimental mixing points distributed along them. The number of mixing point nodes was dictated by the relative length of the tie lines. Thus, a network of experimental points were created, which served as a base for laccase concentration prediction. Basing on measured activities of both phases and their respective volumes, concentration factors (Eq. 3), yields (Eq. 4), and volume fractions (Eq. 5) were calculated.

To achieve high reliability of the empirical short-cut method, each of the five sectors of the two-phase range were approximated separately. The values of experimental concentration factors were interpolated with a biharmonic interpolation function in MATLAB^®^.

Knowing the values of concentration factors, thanks to the empirical stage and the volume fractions, thanks to the equilibrium stage, a value of bottom phase yield could be calculated (Eq. 14).

14 $${\text{CF}}_{\text{BP}} \cdot {\text{VF}}_{\text{BP}} = \frac{{{\text{act}}_{\text{BP}} }}{{{\text{act}}_{\text{tot}} }} \cdot \frac{{V_{\text{BP}} }}{{V_{\text{tot}} }} = \frac{{{\text{act}}_{\text{BP}} \cdot V_{\text{BP}} }}{{{\text{act}}_{\text{tot}} \cdot \frac{{V_{\text{tot}} }}{{V_{\text{SN}} }} \cdot V_{\text{SN}} }} = \frac{{{\text{act}}_{\text{BP}} \cdot V_{\text{BP}} }}{{{\text{act}}_{\text{SN}} \cdot V_{\text{SN}} }} = Y_{\text{BP}}$$

Thus, the proposed method is able to predict the tie line equation, the phase equilibrium composition of the phases, the mass and volume fractions of the phases, the bottom phase concentration factor of laccase, and the bottom phase yield of laccase with the mixing point composition as the only input variable.

## Results and discussion

A set of batch experiments at different mixing points was carried out to gather information for the empirical stage. Each experiment was performed in triplicate, and the average values of CF were treated as input. Average concentration factor values along with their corresponding mixing points and standard deviations are presented in Table 3. It can be noticed that mixing points on longer tie lines, such as tie line A (mixtures with high amounts of phase forming components), correspond to relatively low concentrations in the bottom phase. Additionally, mixing points corresponding to low-bottom phase volume fractions (Mixtures with low relative content of phosphate, such as experiment nos. 18, 22, 25) gave relatively high concentration factors because the majority of the enzyme was concentrated within a small volume. Both phenomena were described in the previous work (Blatkiewicz et al. 2016). It can also be noticed that such points were encumbered with relatively high measurement errors. It can be explained by the fact that very small differences in preparation of the mixtures in proximity to the binodal curve can lead to significant changes in phase volumes, thus effecting in deviations between experiments performed in triplicates.

**Table 3:** Mixing points and their corresponding concentration factor values for batch experiments

Exp no.	Tie line	PEG [wt%]	Phos. [wt%]	CF [UL^−1^/UL^−1^]	Exp no.	Tie line	PEG [wt%]	Phos [wt%]	CF [UL^−1^/UL^−1^]
1	A	39.7	3.1	0.515 ± 0.021	15	C	22.0	6.7	1.499 ± 0.012
2	A	33.1	5.6	0.543 ± 0.117	16	C	13.0	9.8	0.987 ± 0.102
3	A	26.5	8.1	0.245 ± 0.024	17	C	6.5	12.0	0.734 ± 0.106
4	A	19.8	10.6	0.192 ± 0.028	18	D	27.3	3.3	5.391 ± 0.121
5	A	13.2	13.1	0.178 ± 0.027	19	D	20.5	5.5	2.156 ± 0.070
6	A	6.6	15.6	0.257 ± 0.017	20	D	13.7	7.8	1.514 ± 0.020
7	B	36.6	2.9	1.183 ± 0.129	21	D	6.8	10.0	1.124 ± 0.004
8	B	30.5	5.1	1.070 ± 0.049	22	E	21.5	3.6	4.670 ± 0.162
9	B	24.4	7.3	0.600 ± 0.027	23	E	14.5	5.8	1.954 ± 0.004
10	B	18.3	9.5	0.495 ± 0.017	24	E	7.4	8.0	1.032 ± 0.047
11	B	12.2	11.7	0.491 ± 0.028	25	F	15.0	4.0	5.474 ± 0.162
12	B	6.1	13.9	0.506 ± 0.030	26	F	10.0	5.3	1.403 ± 0.063
13	C	32.4	3.1	3.982 ± 0.111	27	F	5.0	6.4	0.962 ± 0.009
14	C	25.9	5.3	2.061 ± 0.049					

It is important to choose mixture compositions for continuous extraction relatively far away from the critical point and the binodal curve in general since small disturbances in the feed could lead to local transitions into a one-phase system. Several “safe” mixing points chosen for extraction in the mixer settler unit were presented in Table 4.

**Table 4:** Mixing points for continuous extraction in a mixer-settler unit

Exp. no.	PEG conc. [wt%]	Phos. conc. [wt%]	Phos. solution flow [g/s]	PEG solution flow [g/s]	Supernatant flow [g/s]
1	23.5	4.6	0.030	0.010	0.023
2	7.8	9.2	0.009	0.018	0.030
3	34.7	5.4	0.038	0.010	0.006
4	14.3	12.6	0.018	0.027	0.017
5	27.5	3.3	0.034	0.007	0.020
6	17.0	8.1	0.021	0.017	0.023

**Fig. 5:**
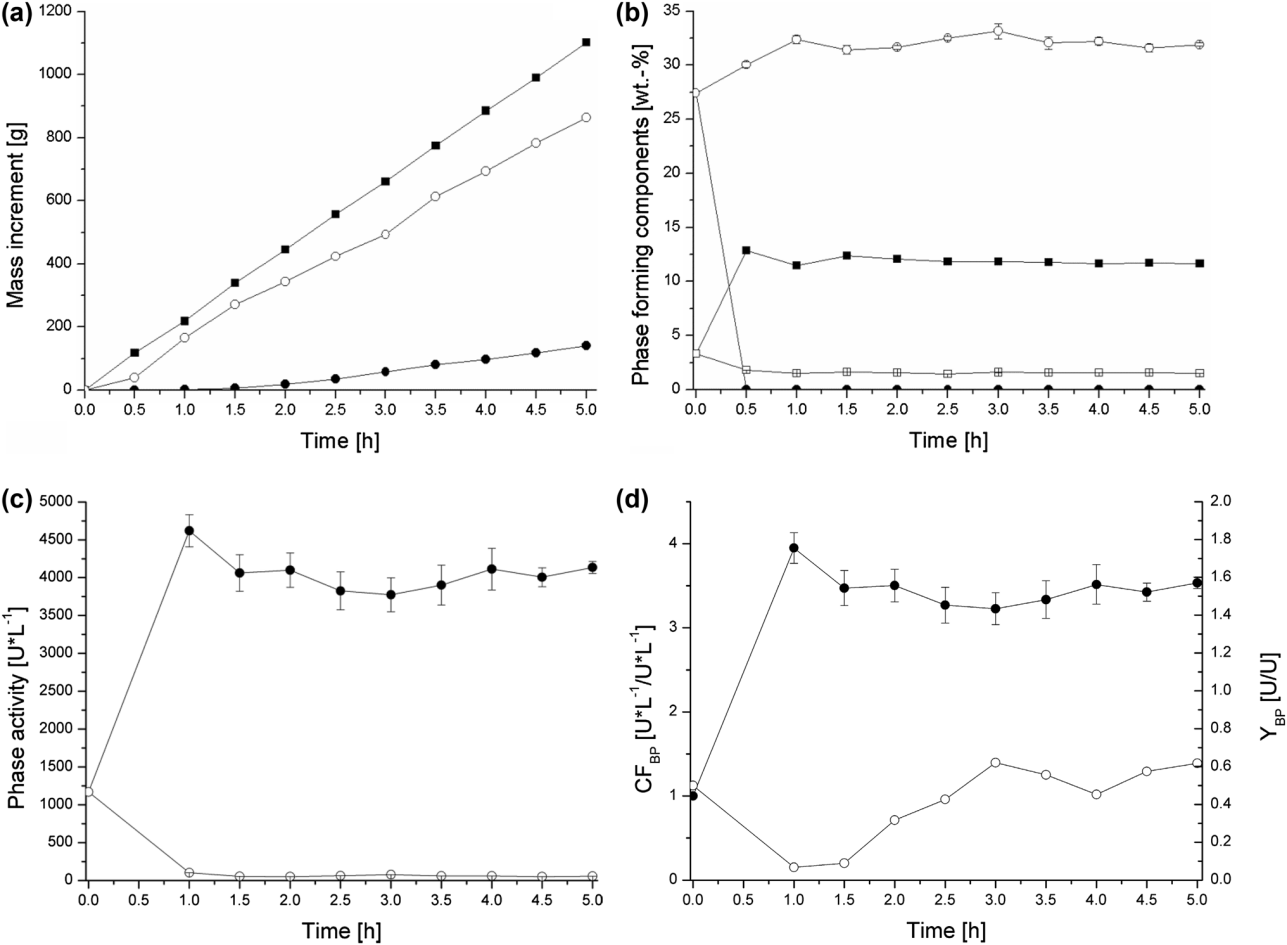
**a** Mass increments of substrates and products over time: sum of substrates (filled square), mass of bottom phase (filled circle), mass of top phase (open circle). **b** Phase-forming components in phases over time: phosphate in bottom phase (filled square), PEG in bottom phase (filled circle), phosphate in top phase (open square), PEG in top phase (open circle). **c** Laccase activities in phases over time: bottom phase (filled circle), top phase (open circle). **d** Efficiency parameters over time: bottom phase concentration factor (filled circle), bottom phase yield (open circle)

An example (experiment no. 5, see Table 4) of continuous extraction of *C. unicolor* laccase was shown in Fig. 5, where changes in process parameters over time were drawn. The flows of stock solutions and culture supernatant were set in a way that the volume of the settler part was exchanged within an hour. However, steady state of the process could be achieved only after the product flows, controlled manually with the spillway window, were established. In this case, the steady-state was achieved after 3 h of the process (Fig. 5a). The following experimental points served to calculate mass flows of substrates and products according to Eq. (7), and the mass balance (Eq. 8) was evaluated. Because the steady state of the process was defined by the steady state of the product flows, the average values of other parameters were calculated from the same corresponding experimental points; in this case, the final four.

The phases separated quickly. After about an hour the phases reached equilibrium and maintained similar results for the rest of the process (Fig. 5b). As expected from the experimental binodal curve (Fig. 3a), PEG 6000 content in the bottom phase was below the detectability threshold, while the phosphate content in the top phase stabilized at about 1.5 wt%.

From the data in Fig. 5c it can be concluded that the chosen mixing point is effective for laccase concentration. The enzymatic activity in the bottom phase quickly reached over 4000 U/L, and remained between 3800 and 4200 U/L for the remaining time of the process. At the same time, the activity of laccase in the top phase did not exceed 100 U/L. The calculated parameters were presented in Fig. 5d. The bottom phase concentration factor remained around 3.5 for the majority of the process, while the average bottom phase yield was about 0.65.

**Fig. 6:**
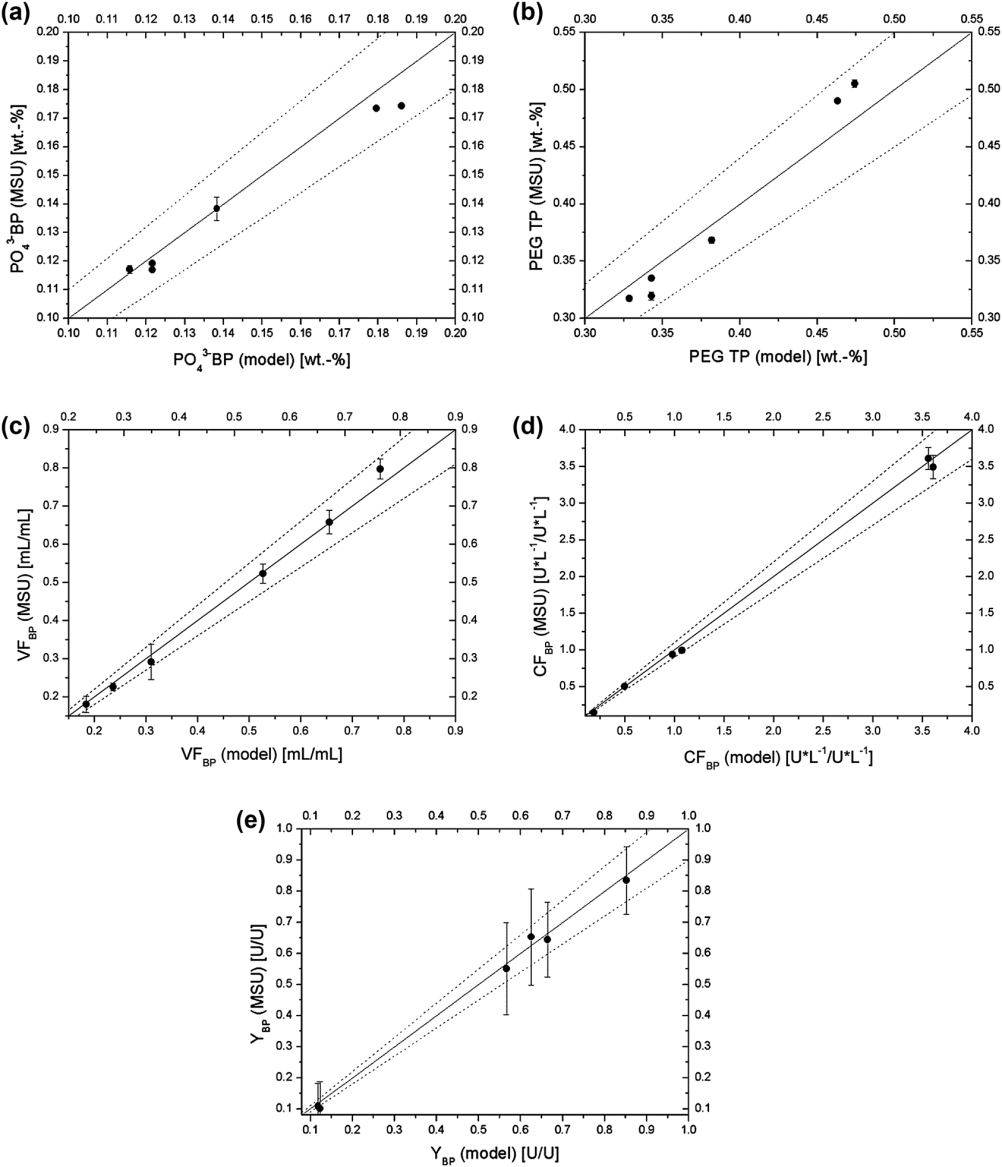
Parity plots of experimental and theoretical values:** a** phosphate content in bottom phase, **b** PEG 6000 content in top phase, **c** bottom phase volume fraction, **d** bottom phase concentration factor, **e** bottom phase yield

The remaining continuous extraction experiments were performed analogously. The resulting data were compared to simulated values in the form of parity plots (Fig. 6).

The parity plots included phosphate content in bottom phase (Fig. 6a), PEG 6000 content in top phase (Fig. 6b), bottom phase volume fraction (Fig. 6c), bottom phase concentration factor (Fig. 6d), and bottom phase yield (Fig. 6e). PEG 6000 content in bottom phase and phosphate content in top phase were not included, as the experimental results significantly differed from the simulated values. It was caused by limited separation time in the settler, especially in the case of top phase, which was significantly more viscous due to high polymer content.

All the experimental values lay within the ± 10% error range of simulated values. Although encumbered with high errors, caused by multi-step Gaussian propagation, the enzymatic yield values also fitted within the error range. It can be noticed that there are differences in fitting accuracy between different parameters. For example, volume fraction data points are very similar to the short-cut method’s predictions (Fig. 6c), while phase composition data points (Fig. 6a, b) showed more significant deviations. Since the mixture flow throughout the equipment was relatively high (220 g/h), the mass measurements were very accurate, which lead to high similarity between the experimental and the simulated values. At the same time, the phase compositions could not be measured with comparatively high accuracy, due to limited separation time of the phases along with two-step dilution for the chromatographic analyses, which may propagate the measurement error. Because the chosen mixing points for the continuous experiments lay away from the binodal curve, the respective bottom phase concentration factors (Fig. 6d) were encumbered with relatively low measurement errors due to comparable settled phase volumes (compare to Table 3).

## Conclusions

The aqueous two-phase system consisting of PEG 6000 and pH 7 phosphate buffer is suitable for continuous single-step concentration of *C. unicolor* laccase within a mixer-settler unit. An hour-long retention time in the settler provides enough time for separation of the phases for collection. Both the tie-line length and the phase–volume ratio have substantial effect on enzyme concentration and yield.

The proposed short-cut method provides effective predictions of the system’s behavior in a steady state, including the phase equilibria and extraction efficiency parameters of *C. unicolor* laccase concentration. The predictions are in a good accordance with the experimental data. It was discovered that elongated tie lines of the PEG 6000—phosphate system converge in one common point, which creates a convenient method of tie line interpolation, but it has to be supported by evidence that it also works with other aqueous two-phase systems.

Although the PEG 6000—phosphate ATPS provides high enough extraction efficiency for only one-step extraction process, the method can be extended to multi-step extraction for other biological compounds.

## Symbols

act: Enzymatic activity, U L^−1^
*B*: Ion to salt mass ratio constant
*b*: Binodal curve regression parameter
*CF*: Concentration factor
*D*: Dilution factor
*d*: Optical length, cm
*E*: Absorbance
En: Enrichment
*m*: Mass, g
*M*: Mass fraction
*t*: Time, min
*V*: Volume, mL
VF: Volume fraction
*Y*: Enzymatic yield
*x*: Mass fraction

## Greek letters

*ε*_*420*_: Extinction coefficient at 420 nm, L mmol^−1^ cm^−1^
*ρ*: Density, kg m^3^

## Subscripts

BP: Bottom phase
H2O: Water
M: Mixing point
P: Tie line convergence point
PEG: Polyethylene glycol
phos: Phosphate
s: Sample
SN: Supernatant
sub: Substrates
TP: Top phase
tot: Total
